# Chloroplast Genome Sequence of Snap Bean (*Phaseolus vulgaris* L.) and Comparative Analyses with Other Legume Chloroplast Genomes

**DOI:** 10.3390/ijms27188393

**Published:** 2026-09-20

**Authors:** Zhiyuan Liu, Jiawei Wang, Zhaosheng Xu, Helong Zhang, Hongbing She, Wei Qian

**Affiliations:** State Key Laboratory of Vegetable Biobreeding, Institute of Vegetables and Flowers, Chinese Academy of Agricultural Sciences, Beijing 100081, China

**Keywords:** snap bean, chloroplast genome, comparative analysis, phylogenetic analysis

## Abstract

Common bean (*Phaseolus vulgaris* L.) is an economically important grain legume for the human diet and sustainable agricultural development. Snap bean, a vegetable-type common bean cultivated for edible immature pods, serves as a crucial dietary source of essential vitamins, minerals and proteins. However, its evolutionary and domestication history remains poorly characterized. Here, we assembled and characterized the complete chloroplast genome of snap bean using PacBio HiFi sequencing technology. The chloroplast genome formed a single circular molecule of 150,248 bp, consisting of a pair of inverted repeat regions, a large single-copy region, and a small single-copy region. A total of 75 protein-coding genes, 37 tRNA genes, and 8 rRNA genes were annotated. Phylogenomics revealed that snap bean has the closest relationship with dry common bean, grouping with lima bean; *Phaseolus* is phylogenetically nearest to *Vigna*. Comparative plastid genomics identified variants in *accD*, *rpoC2*, *matK*, and other coding genes. Nucleotide diversity analysis detected several highly variable hotspot regions, including *accD*, trnY-psbD, ndhH, and ycf1-rps15, which are promising informative plastid markers for phylogeny and population genetics. These findings deepen our understanding of legume chloroplast evolution and lay genomic foundations for common bean phylogenetic research and genetic breeding improvement.

## 1. Introduction

Common bean (*Phaseolus vulgaris* L.) represents one of the five domesticated species within the genus Phaseolus, alongside tepary bean (*P. acutifolius* A. Gray), runner bean (*P. coccineus* L.), lima bean (*P. lunatus* L.) and year-long bean (*P. dumosus* Macfad.) [1]. It is characterized by broad adaptability; convenient cultivation; and being an important source of protein, vitamins and micronutrients. Meanwhile, it also serves as an ideal model plant to dissect adaptive differentiation between wild and domesticated legume populations. Many studies have confirmed that wild common bean populations have diverged into three independent gene pools, namely the Mesoamerican gene pool, the Andean gene pool, and the northern Peru-Ecuador gene pool [2,3]. Long-term domestication of common bean has given rise to two principal consumption forms. Dry beans are harvested for mature seeds and widely eaten as staple foods, whereas snap beans are cultivated to provide tender immature pods for fresh vegetable consumption. Some studies hypothesized that snap beans were derived from dry beans [4], but reliable molecular proof to verify this evolutionary relationship remains limited.

Chloroplast genomes exclusively exist in plants and algae [5]. Chloroplasts are indispensable semi-autonomous organelles in plant cells, acting as the primary functional sites for photosynthesis, mediating cytoplasmic inheritance, and regulating plant immune responses [6]. Chloroplasts and mitochondria are thought to have arisen from independent endosymbiotic events dating back more than 1.5 billion years [7]. Given the maternal inheritance pattern of chloroplast genomes in angiosperms, these genomic data are widely adopted to conduct phylogenetic analyses and population genetic research [8,9]. Chloroplast genomes of land plants are highly conserved, consisting of circular double-stranded DNA molecules with a size of 120–160 kb and encoding roughly 110–150 functional genes [10]. A typical conserved quadripartite architecture is observed in most chloroplasts, where a pair of inverted repeat (IR) regions divide the whole genome into one large single-copy (LSC) region and one small single-copy (SSC) region [11]. Nevertheless, chloroplast genomes still harbor abundant sequence polymorphisms and structural gene rearrangements, which are mainly driven by the expansion and contraction of inverted repeat boundaries [12]. Such genomic variations generate valuable phylogenetic signatures and exert vital impacts on studies of plant evolutionary trajectories. Benefiting from their compact molecular size, conserved structural features, and relatively stable gene composition, chloroplast genomes serve as ideal research materials for comparative and evolutionary genomics, which favors phylogenetic tree reconstruction and the exploitation of molecular markers [13,14,15].

As the publicly available chloroplast genomic resources for the tribe *Phaseoleae* species have increased rapidly over recent years, chloroplast genome sequences of several common bean accessions have been reported and characterized [16,17]. These datasets provided valuable yet still limited information on evolution and domestication. Regrettably, most studies predominantly focus on dry common bean germplasm resources, whereas the chloroplast genome for the snap bean is still lacking, and the chloroplast genome divergence between the snap bean and dry bean has not yet been directly evaluated. Therefore, this study performs whole chloroplast genome assembly and functional annotation for a snap bean accession and conducts a comprehensive comparative genomic analysis with other published chloroplast genomes within *Phaseoleae*. The results are expected to deepen our understanding of legume crop evolution and promote the exploitation of snap bean as a valuable genetic resource for bean crop improvement.

## 2. Results

### 2.1. Characteristics of the Snap Bean Chloroplast Genome

**Figure 1:**
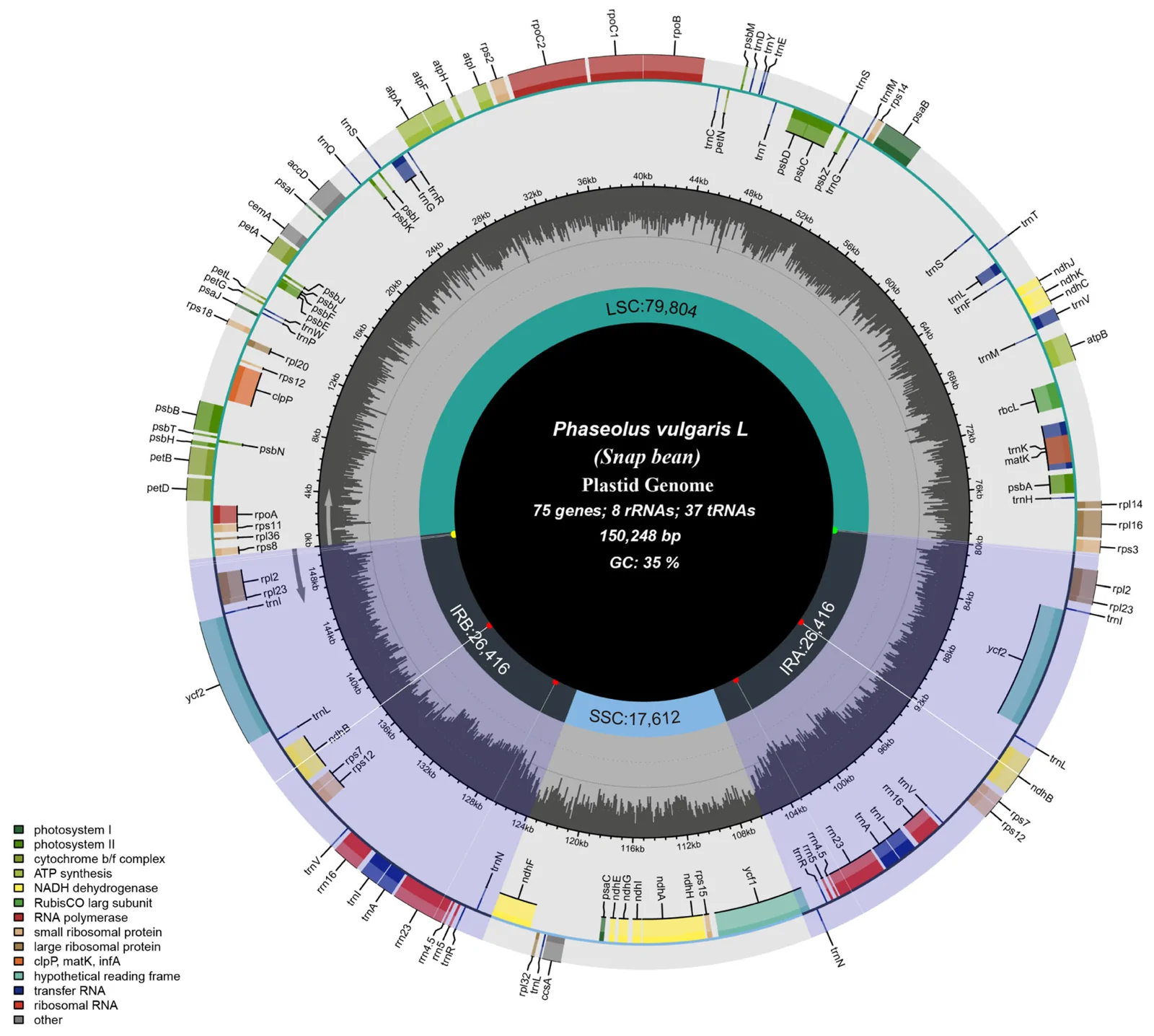
The chloroplast genome structure of snap bean. From the center outward, the first track shows the large single-copy(LSC), inverted repeat(IRA and IRB), and small single-copy(SSC). The GC content along the genome is plotted on the second track. The genes are shown on the third track. The transcription directions for theinner and outer genes are clockwise and anticlockwise, respectively. The functional classification of the genes is shown in the bottom leftcorner.

The complete chloroplast genome of the snap bean was assembled into a circular DNA molecule of 150,248 bp and exhibited a typical quadripartite structure. This structure comprises a pair of inverted repeat regions (IRa and IRb, each 26,416 bp), a large single-copy region (LSC) (79,804 bp) and a small single-copy region (SSC) (17,612 bp) (Figure 1). The overall GC content of the chloroplast genome was about 35%, which is consistent with that of other legume chloroplast genomes [18]. The GC contents of the LSC, SSC, and IR regions were 33%, 28%, and 42%, respectively (Table 1).

**Figure 2:**
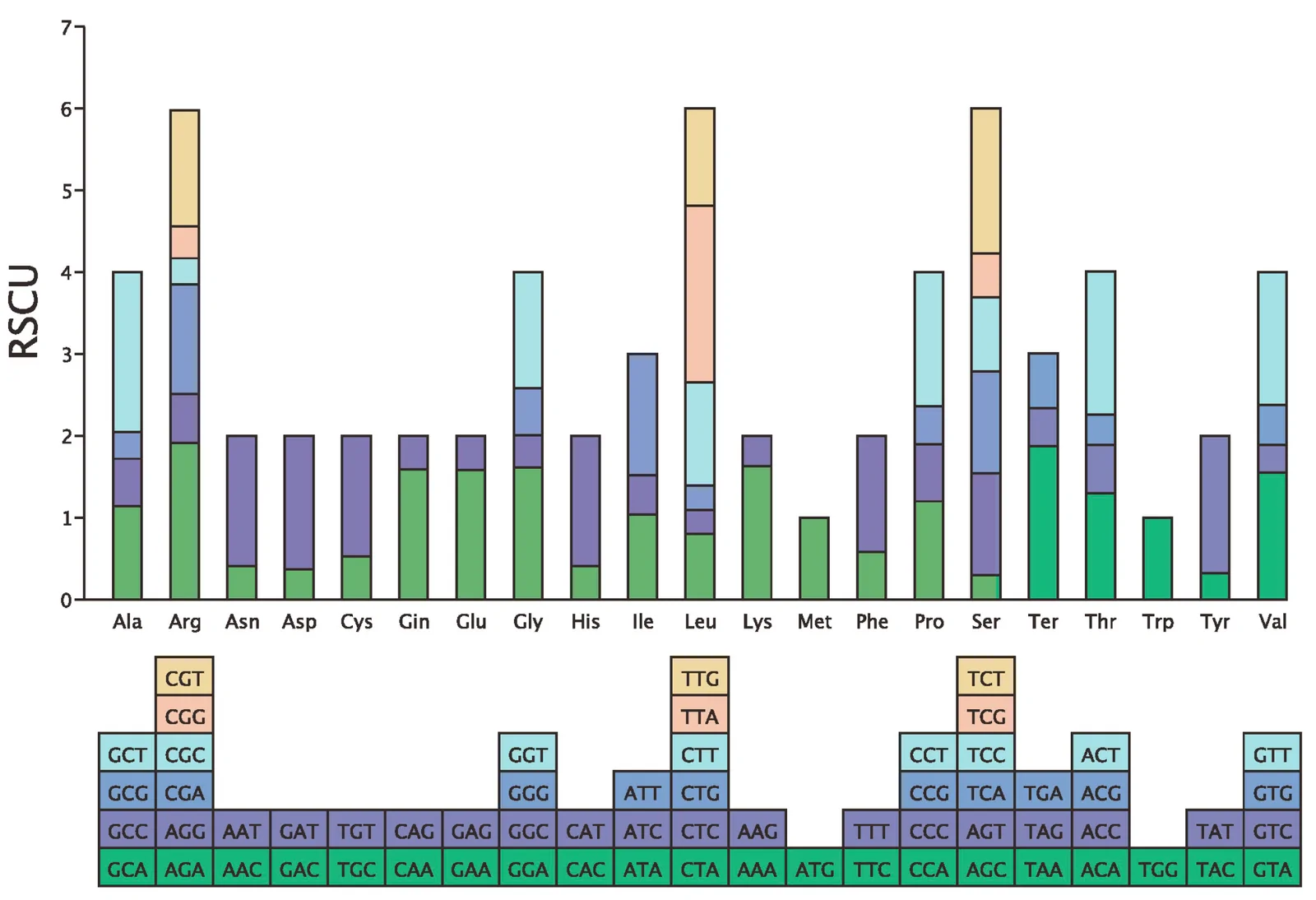
Relative Synonymous Codon Usage (RSCU) of amino acids in the Cp genome. The height of each bar represents the RSCU value for the corresponding amino acid. The colors within the bar chart indicate different codons.

A total of 120 genes were annotated, consisting of 75 protein-coding genes, 37 tRNA genes, and 8 rRNA genes (Table 2). Specifically, 75 genes were distributed in the LSC region, including 53 protein-coding genes and 22 tRNA genes; 13 genes were assigned to the SSC region, and the remaining 32 genes resided within the IR repeat region, where all eight rRNA genes were exclusively localized. We subsequently calculated the codon usage profiles of these protein-coding genes, which were constituted by a total of 19,585 codons (Table S1). Codon usage bias analysis clearly revealed a strong bias toward A/U-ending codons, agreeing with the typical AT-rich genomic composition. In addition, leucine, serine, and arginine were the most prevalent amino acids (Figure 2).

Overall, we identified 16 intron-containing genes across the snap bean chloroplast genome, consisting of 10 protein-coding genes and six tRNA genes (Table 2). Among these, 14 genes carried a single intron, whereas two genes (*clpP* and *rps12*) possessed two introns. There are 16 pairs of duplicated genes, with four pairs of rRNA genes located in the IR repeat region.

### 2.2. Phylogenetic Analysis of Chloroplast Genomes

**Figure 3:**
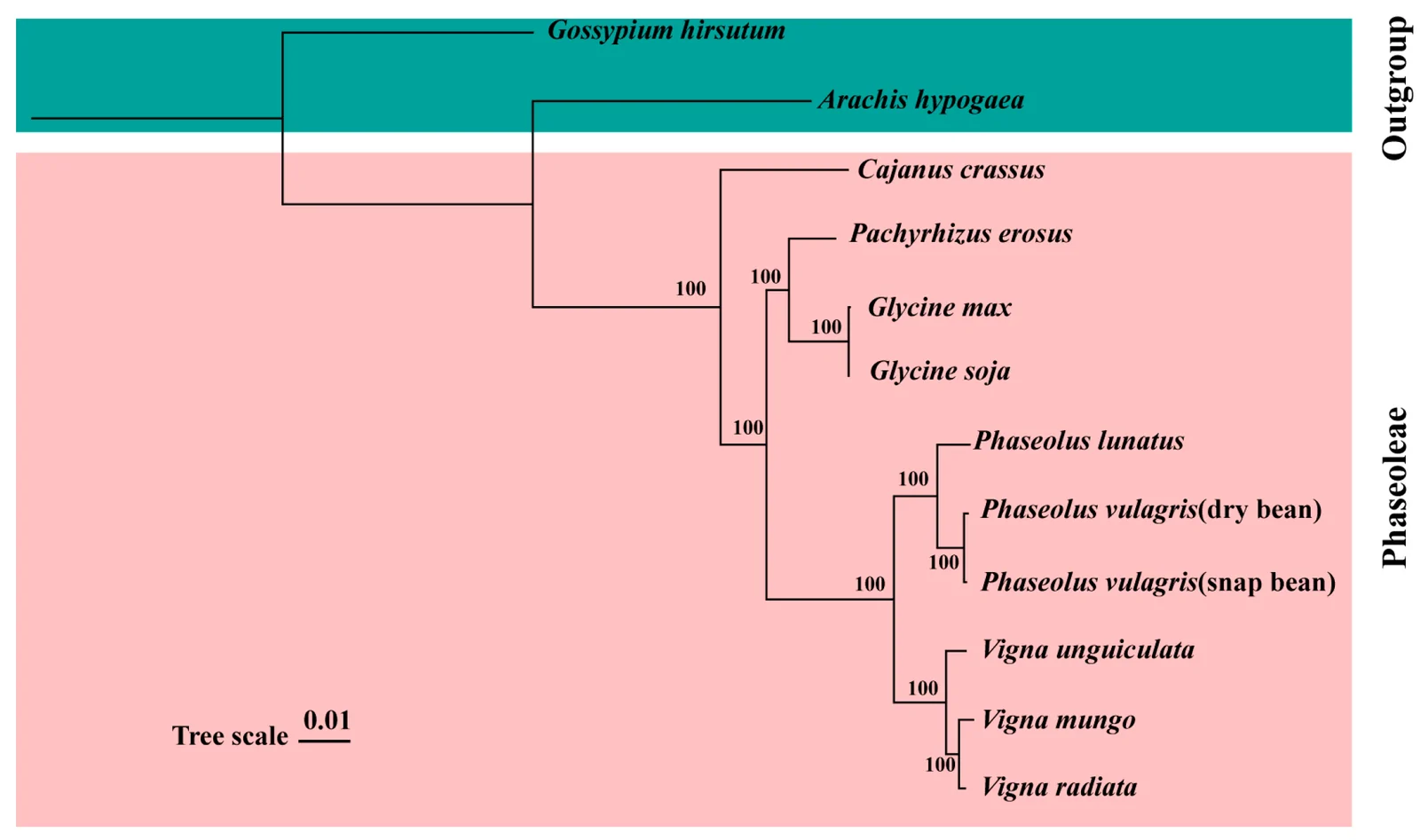
Phylogenetic tree of 12 legume species based on chloroplast genome. Ultrafast bootstrap support values (1000 replicates) are shown on branches. The scale bar represents the number of nucleotide substitutions per site.

To investigate the phylogenetic relationships, a maximum likelihood (ML) phylogenetic tree was reconstructed based on chloroplast genome sequences of 10 *Phaseoleae* species, including *Cajanus crassus*, *Glycine max*, *Glycine soja*, *Pachyrhizus erosus*, *Phaseolus lunatus*, *Phaseolus vulgaris* (dry bean and snap bean), *Vigna unguiculata*, *Vigna mungo*, and *Vigna radiata*. *Arachis hypogaea* and *Gossypium hirsutum* were designated as outgroups (Figure 3). Snap bean and dry bean are two divergent domesticated morphotypes of *Phaseolus vulgaris*. Together with lima bean (*Phaseolus lunatus*), they clustered into a compact monophyletic clade within the genus *Phaseolus*. The *Phaseolus* clade exhibited the closest phylogenetic affinity to the genus *Vigna*, which comprises three economically important crops: mung bean (*Vigna radiata*), black gram (*Vigna mungo*) and cowpea (*Vigna unguiculata*). Cultivated soybean (*Glycine max*) derived from wild soybean (*Glycine soja*) bears highly similar genetic compositions, yet the *Glycine* lineage diverged earlier than the *Phaseolus* and *Vigna* clades. *Cajanus crassus* corresponds to a basal group in this tribe and has an earlier evolutionary separation relative to *Phaseolus* (Figure 3).

### 2.3. Long Repeats Sequence and Simple Sequence Repeats Analysis

**Figure 4:**
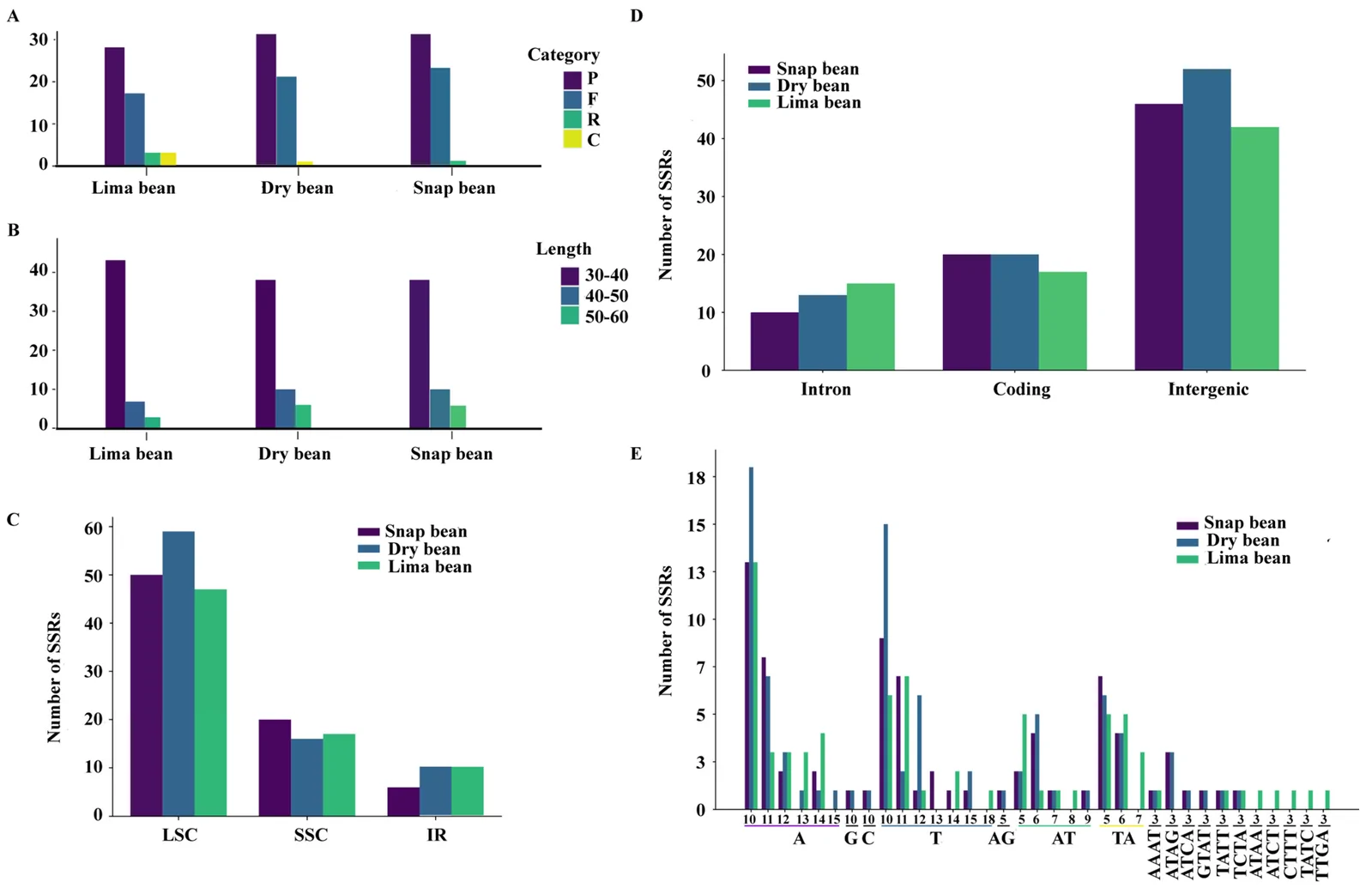
Repetitive sequences in the chloroplast genomes of three *Phaseolus* species. (**A**) Number of different long repeat types. (**B**) Length distribution of the Phaseolus species. (**C**) The proportion of SSRs (Simple Sequence Repeats) in the LSC (Large Single Copy), SSC (Small Single Copy), and IR (Inverted Repeat) regions. (**D**) Distribution of SSRs in intron, coding, and intergenic regions. (**E**) Analysis of SSRs in different-length repetitive sequences and the distribution of repetitive sequences.

Long repetitive sequences (≥30 bp) were annotated via the REPuter software across lima bean, dry bean and snap bean. A total of 51, 53 and 53 repeat pairs were identified in the three taxa, respectively (Table S2). A total of 28, 31 and 31 palindromic repeats, alongside 17, 21 and 21 forward repeats, were separately detected in lima bean, dry bean and snap bean. Furthermore, three complementary repeats were detected in lima bean and one in dry bean. No reverse repeats were identified in dry bean, while three reverse repeats were present in lima bean and one in snap bean (Figure 4A). The length distribution of long repeats from these three beans was predominantly clustered within the 30–40 bp interval, while repeats ranging from 50 to 60 bp were extremely scarce (Figure 4B). Intriguingly, the overall size distribution and main categories (palindromic and forward repeats) of long repeats were highly similar between dry bean and snap bean.

To characterize the distribution patterns and composition features of simple sequence repeats (SSRs) across the chloroplast genomes of three *Phaseolus* beans, we identified a total of 74, 85 and 76 SSR loci in lima bean, dry bean and snap bean, respectively (Table S3). These SSRs were predominantly distributed in the LSC region. Notably, the IR region of snap bean harbored fewer SSRs relative to the other two bean species (Figure 4C). Genomic position distribution revealed that SSRs of the three beans were mostly enriched in intergenic spacers, followed by coding sequences; by contrast, merely 10, 13, and 15 SSRs were distributed within intronic regions (Figure 4D). Mononucleotide SSRs made up 63%, 66.8%, and 58% of the total SSRs in snap bean, dry bean, and lima bean, respectively (Table S3). Multiple polymorphic SSR motifs were detected across the three taxa. Specifically, four motifs, including (AAAT)_3_, (GTAT)_3_, (TATT)_3_ and (TCTA)_3_, were common in all three beans, whereas (ATAG)_3_ and (ATCA)_3_ existed exclusively in snap bean and dry bean. Additionally, the five motifs (ATAA)_3_, (ATCT)_3_, (CTTT)_3_, (TATC)_3_ and (TTGA)_3_ were unique to lima bean and undetectable in snap bean and dry bean (Figure 4E).

### 2.4. Comparative Analysis of Complete Chloroplast Genomes

**Figure 5:**
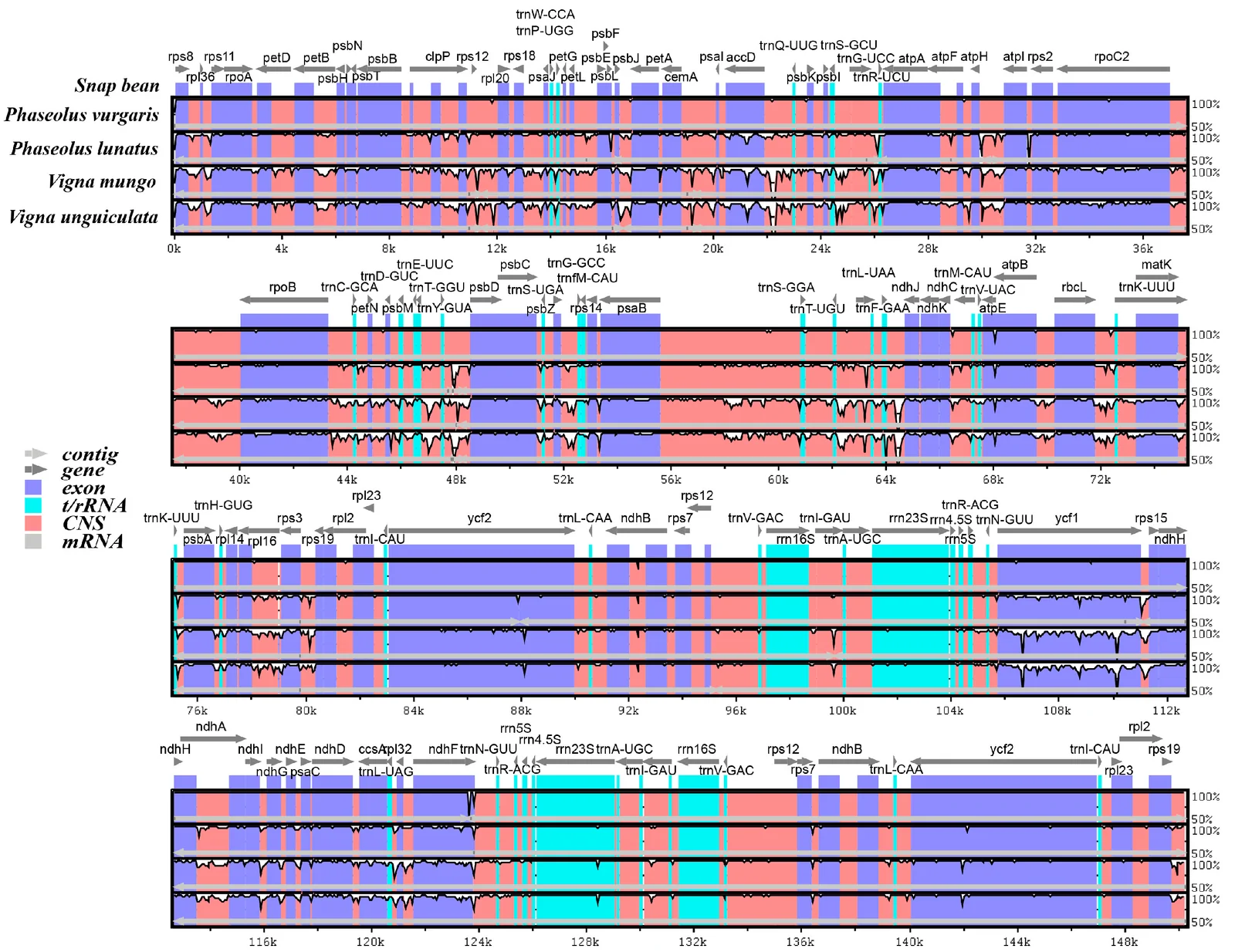
Comparison of the chloroplast genomes of five legume species using mVISTA. The gray arrows above the alignment indicate the direction of gene transcription. The *y*-axis represents the percentage of identity ranging from 50% to 100%. Protein-coding genes (exons), rRNA, tRNA, and conserved non-coding sequences (CNS) are displayed in different colors.

To further characterize chloroplast genomic variations, we conducted a genome-wide comparison of chloroplast genome sequences from *Phaseolus* and *Vigna* clades using mVISTA software, with the snap bean genome as the reference sequence (Figure 5). Genome-wide alignment revealed nearly identical genomic sequences between dry bean and snap bean. Nevertheless, several coding genes (*accD*, *rpoC2*, *matK*, *ndhF*, *ndhH* and *ycf1*) exhibited obvious sequence variations across *P. lunatus*, *V. mungo*, and *V. unguiculata*.

Meanwhile, multiple intergenic spacers, including rps12-rpl20, rps8-rpl36, accD-psbK, psbI-trnR, trnY-psbD, trnF-ndhJ, ycf1-rps15 and rbcL-trnK, showed high divergence in non-coding regions. Collectively, these sequence variations are presumed to result from differential environmental selection during the evolutionary process of *Phaseolus* and *Vigna* clades, which lays a foundation for exploring their adaptive evolution.

### 2.5. Selection Pressure Analysis

**Figure 6:**
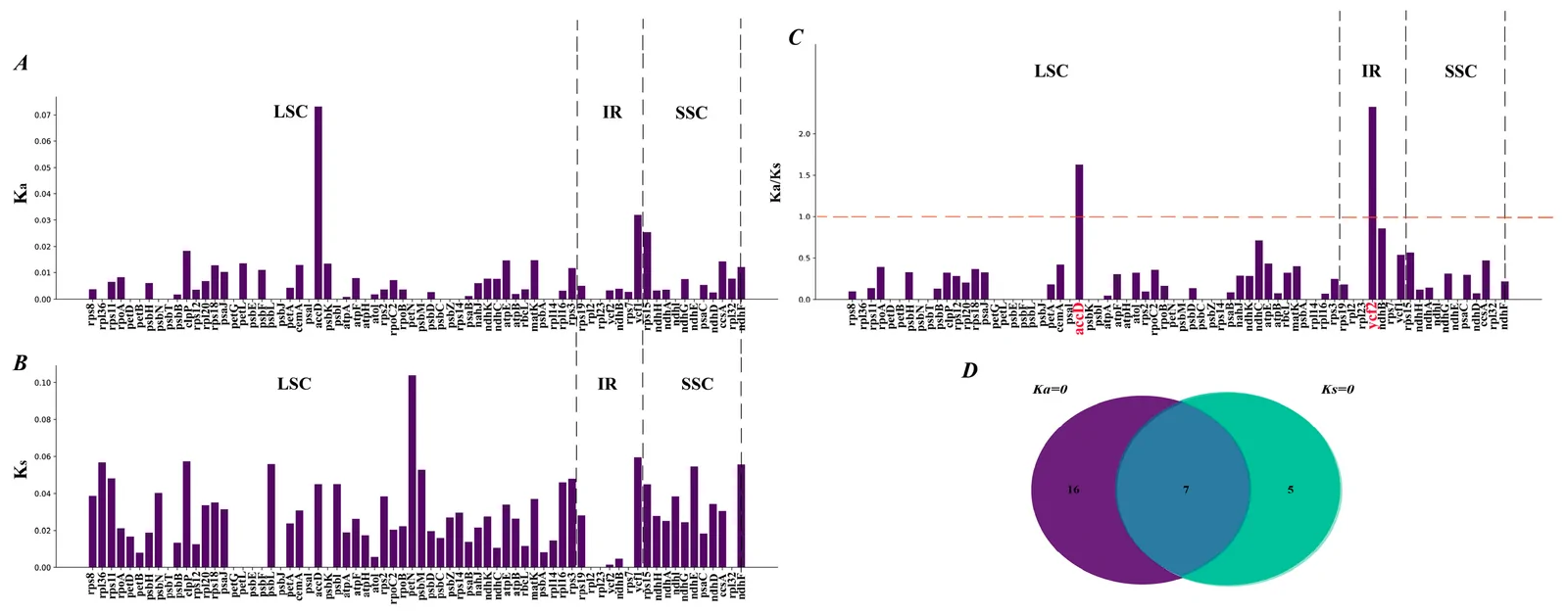
Non-synonymous (Ka), synonymous (Ks), and Ka/Ks substitution values of snap bean and lima bean. (**A**) Ka. (**B**) Ks. (**C**) Ka/Ks substitution values. (**D**) Venn diagram of protein-coding genes with Ka = 0 and Ks = 0. The genes highlighted in red were under positive selection during the evolution of *Phaseolus*. The red dashed line indicates a Ka/Ks value of 1.

To assess selective pressure among three beans, we calculated the nonsynonymous substitution rate (Ka) and synonymous substitution rate (Ks), as well as Ka/Ks ratios, based on 70 homologous gene pairs. Only nine variable sites were detected in the protein-coding regions between dry bean and snap bean, resulting in undetermined Ka and Ks values for most genes (Figure S1). In contrast, the Ka values between snap bean and lima bean ranged from 0.0000 to 0.0731, while the Ks values ranged from 0.0000 to 0.1038 (Figure 6A,B; Table S4). Furthermore, a total of 23 genes exhibited a Ka value of zero and 12 genes exhibited a Ks value of zero between snap bean and lima bean. Among them, seven genes had both Ka and Ks values of zero, indicating extreme sequence conservation of these genes during the evolutionary process of lima bean (Figure 6D). Among the 70 protein-coding genes, 12 genes were excluded from Ka/Ks calculation due to a Ks value of zero. Of the remaining 58 genes, 16 (28%) exhibited a Ka/Ks ratio of zero, and 42 (72%) had a Ka/Ks ratio greater than zero. Notably, only two genes were identified under positive selection: the *accD* gene located in the LSC region and the *ycf2* gene in the IR region (Figure 6C).

**Figure 7:**
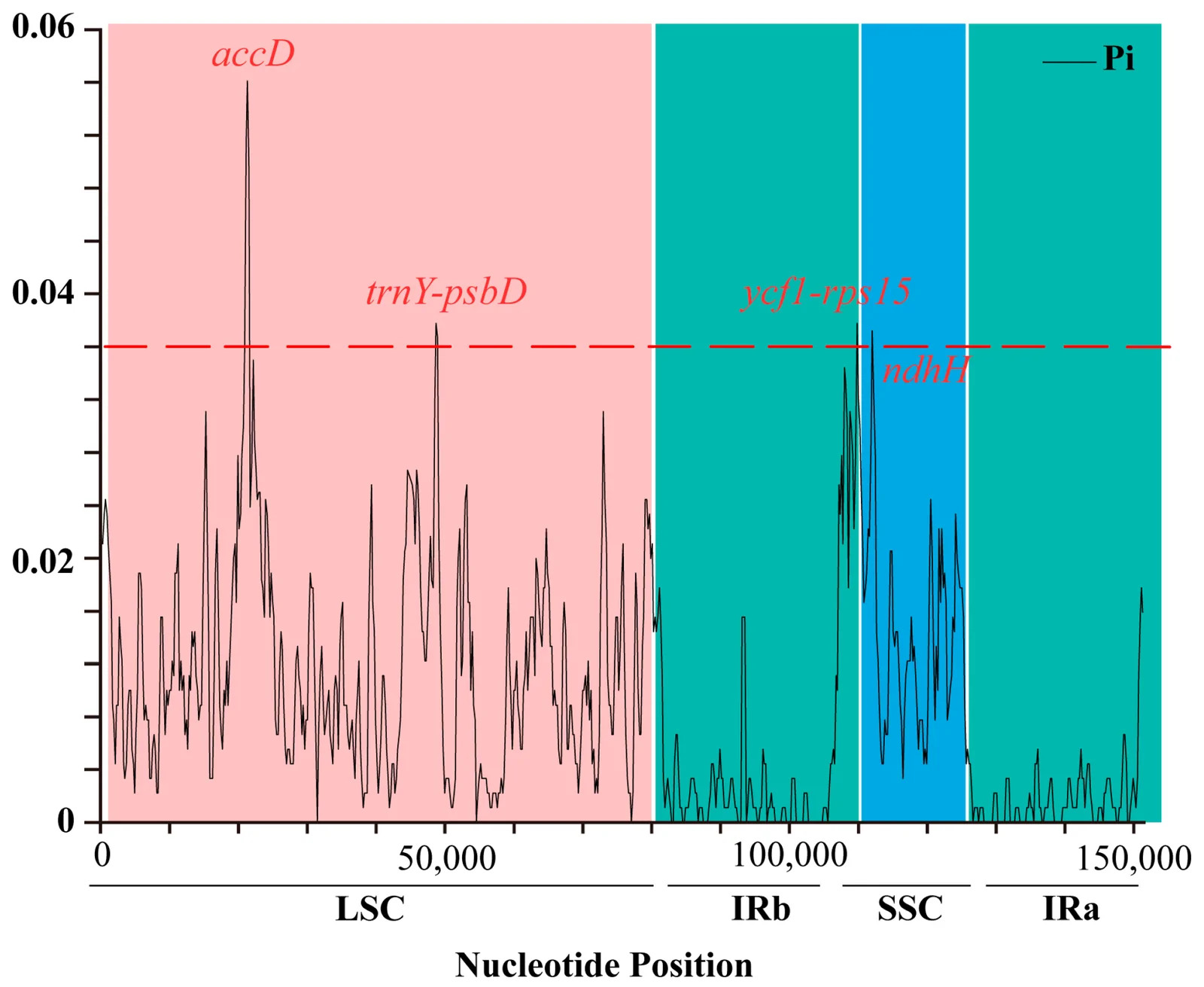
Nucleotide diversity (Pi) analysis of chloroplast genomes in Phaseolus species. Pi was calculated with 600 bp sliding windows and a 200 bp step size. The red dashed line indicates a Pi value of 0.035.

Sliding-window analysis of nucleotide diversity (Pi) was performed to evaluate the overall genetic variation across the chloroplast genome of *Phaseolus* beans. Several highly variable mutation hotspots were identified. The *accD* gene, located in the large single-copy (LSC) region, exhibited the highest variability (Pi > 0.05) (Table S5). In addition, three other genes (*trnY-psbD*, *ndhH* and *ycf1-rps15*) showed moderate diversity (Pi > 0.035) (Figure 7).

## 3. Discussion

In this study, we assembled and annotated the complete chloroplast genome of a snap bean accession using high-accuracy PacBio HiFi reads and performed comparative genomic analysis with published dry bean and other Phaseoleae chloroplast genomes. PacBio HiFi sequencing was used to generate high-accuracy long reads for chloroplast genome assembly of this snap-bean accession, ensuring high base-level accuracy across repeat-enriched IR regions [18]. The snap bean chloroplast genome exhibited a typical quadripartite structure with a genome size in the range of 120 kb to 160 kb, and the overall gene content and genomic organization were highly conserved, which were consistent with the fundamental characteristics of previously reported common bean and legume chloroplast genomes [16]. These genomic features indicated that the chloroplast genome sequence of both snap bean and dry bean maintains relatively high structural conservation during long-term domestication and adaptive evolution. In addition, numerous simple sequence repeat (SSR) loci were identified in the snap bean chloroplast genome, dominated by mononucleotide A/T repeats, which is consistent with the universal AT-rich preference of chloroplast genomes in most angiosperms [19,20]. These SSR loci detected in this study provide abundant molecular markers for germplasm identification, genetic diversity evaluation, and population genetic analysis of snap bean [21].

We systematically analyzed and characterized the codon usage patterns of the snap bean chloroplast genome, identifying a total of 19,585 codons. Our results revealed that leucine was the most frequently encoded amino acid and tryptophan the least abundant. Such codon and amino acid preferences substantially influence the efficiency of protein synthesis and the structural and functional characteristics of translated proteins. Notably, leucine is critical for maintaining protein structural stability; it periodically appears every seven residues in numerous DNA-binding proteins and forms the classic “leucine zipper” structure, which facilitates protein dimerization and further regulates downstream biological processes [22]. Furthermore, the synonymous codon usage bias may reflect underlying constraints related to translational efficiency and gene expression [23,24,25]; however, the present study does not directly assess translational efficiency, transcript abundance, or gene expression, and further experimental studies are needed to determine whether codon usage bias modulates chloroplast gene regulation or the adaptive capacity of snap bean.

Genetic diversity of germplasm resources constitutes the core foundation for crop genetic improvement and evolutionary research [26]. In the present study, we identified many sequence variations in protein-coding regions and intergenic regions across snap bean chloroplast genomes. These loci could be utilized as molecular markers to analyze phylogenetic relationships and genetic differentiation among legume species. The *matK* and *ycf1* genes serve as core barcode genes for species delimitation and evolutionary analysis in the genus Meconopsis [27]. The *accD* gene has been repeatedly reported as a hypervariable plastomic region, suitable for low-level phylogenetic inference and DNA barcoding in multiple plants [28]. Similarly, the rpl20-rps12 spacer displays high polymorphism in mango and enables efficient variety identification and phylogenetic analysis [29], while rps8-rpl36 and accD-psbK exhibit significant interspecific variation and are widely applicable for plant species discrimination [30]. Collectively, these plastomic variant loci provide valuable genetic resources for dissecting the evolutionary dynamics and domestication footprints of snap bean.

Non-synonymous (Ka) and synonymous (Ks) substitutions, along with their ratio (Ka/Ks), have been utilized to assess the rates of gene divergence. A Ka/Ks ratio of less than one indicates purifying selection, while a ratio greater than one suggests positive selection [31]. In most protein-coding genes, synonymous nucleotide substitutions occur more frequently than non-synonymous ones. In this study, the majority of genes had a ratio below one, indicating that they are under purifying selection in snap bean. However, the Ka/Ks ratio for the *accD* gene and *ycf2* was greater than one when compared with lima bean, suggesting the two genes may be undergoing positive selection during the evolutionary divergence between *P. vulgaris* and *P. lunatus*.

Chloroplast genomes with high structural conservation and low recombination rates are widely applied in phylogenetic reconstruction and evolutionary research to resolve interspecific phylogenetic relationships and trace domestication origins [32,33,34]. Herein, phylogenetic analyses constructed using whole chloroplast genome sequences deciphered the evolutionary relatedness between snap bean and other Phaseoleae species. The phylogenetic topology revealed that snap bean and dry bean formed a single clade. The extremely low chloroplast genome divergence observed between snap bean and dry bean was consistent with their very close evolutionary relationship. However, because only one snap bean accession was included in this study, morphotype-specific differentiation could not be resolved. More extensive intraspecific sampling is therefore required to further dissect the domestication trajectories of the two morphotypes. Furthermore, the strictly maternal inheritance of the chloroplast genome also constrains our ability to fully elucidate their evolutionary trajectories and genetic differentiation [35,36]. Therefore, integrated large-scale sequencing of chloroplast genomes with nuclear genomes, particularly from wild common bean accessions, is necessary to thoroughly dissect the domestication routes, adaptive variations, and population differentiation profiles of snap bean.

## 4. Materials and Methods

### 4.1. Sequencing and Assembly of Chloroplast Genome

Fresh young leaves of snap bean accession 09B2 were collected in the spring of 2024 from the Institute of Vegetable and Flower Research (IVF), the Chinese Academy of Agricultural Sciences, Beijing. Fresh leaf tissues were immediately frozen in liquid nitrogen for subsequent DNA extraction. High-quality genomic DNA was isolated using a modified CTAB method, and its quality and concentration were assessed through 1% agarose gel electrophoresis and an ND-2000 spectrophotometer (Thermo Fisher Scientific, Wilmington, DE, USA).

Whole-genome sequencing was conducted on the PacBio Sequel II platform adopting PacBio HiFi sequencing technology. Approximately 10 Gb of raw HiFi sequencing data was acquired in this study. Raw long reads were used for de novo assembly with hifiasm software (v 0.25.0-r726) under default parameters. BLASTn (v 2.14.0) was used to align assembled contigs against the published *Phaseolus vulgaris* chloroplast reference genome (EU196765) to screen and extract the complete chloroplast genome sequence of snap bean [37].

### 4.2. Chloroplast Genome Annotation

The online tool GeSeq (available at https://chlorobox.mpimp-golm.mpg.de/geseq.html, accessed on 2 August 2024) was employed to annotate the snap bean chloroplast genome. The circular map of the complete chloroplast genome was visualized with the OGDRAW software (v1.3.1). Calculation of Relative Synonymous Codon Usage (RSCU) values was implemented via CPStools(v 2.0.2) [38], and corresponding RSCU visualization graphs were generated on the BMKCloud bioinformatics platform (http://www.biocloud.net/).

### 4.3. Repeat Structure and Sequence Analysis

The online software REPuter (https://wwww.cebitec.uni-bielefeld.de/bibiserv.cebitec.uni-bielefeld.de/reputer.html, accessed on 10 January 2025) was employed to screen and identify repeat sequences, including forward, reverse, palindromic, and complementary repeats. The screening criteria were defined as a minimum repeat length of 30 bp and a sequence identity threshold above 90%.

Simple sequence repeats (SSRs), encompassing mono-, di-, tri-, tetra-, penta-, and hexanucleotide repeats, were detected via the MISA-web platform (https://webblast.ipk-gatersleben.de/misa/ accessed on 4 March 2025). The thresholds for identifying mono-, di-, tri-, tetra-, penta-, and hexanucleotide repeats were set to 10, 5, 5, 3, 3, and 3, respectively.

### 4.4. Comparative Analysis of Chloroplast Genome

Whole chloroplast genome pairwise alignments were conducted with MAFFT (v7.158b) [39]. The four chloroplast genomes of Phaseoleae species were aligned using mVISTA under the shuffle-LAGAN alignment mode (https://genome.lbl.gov/vista/mvista/about.shtml, accesed on 10 March 2025), where the snap bean chloroplast genome was used as the reference sequence. Nucleotide diversity (Pi) across the chloroplast genomes of *Phaseolus* beans was calculated using DnaSP (v 6.12.03) with a sliding window length of 600 bp and a step size of 200 bp [40].

### 4.5. Adaptive Evaluation Analysis

To estimate the non-synonymous substitution rate (Ka), synonymous substitution rate (Ks), and their corresponding Ka/Ks ratios, we performed pairwise sequence comparisons between snap bean and two *Phaseolus* relatives, namely dry bean and lima bean. Homologous gene sequences from the three species were aligned via MAFFT (v7.158b). Subsequent Ka and Ks values and Ka/Ks ratios were computed using KaKs_Calculator 2.0 [41] with the default model averaging (MA) algorithm. The outputs were visualized with R software (v 4.4.2), and Venn diagrams were produced through the BMKCloud platform.

### 4.6. Phylogenetic Analysis

Whole chloroplast genome sequences of 12 species were used to conduct phylogenetic analysis. *Arachis hypogaea* and *Gossypium hirsutum* were used as outgroups. Of these 12 species, the snap bean was newly sequenced; the other 11 species are available at the National Center for Biotechnology Information (NCBI). Detailed genomic information is in Table S6. The phylogenetic reconstruction was conducted based on whole chloroplast genomes. These sequences were extracted from each genome via CPStools (v 2.0.2). Multiple sequence alignment was conducted using MAFFT (v7.158b) software. A maximum likelihood (ML) phylogenetic tree was built in IQ-TREE2 (v2.1.2), with 1000 ultrafast bootstrap replicates specified by the parameter -bb 1000 [42].

## 5. Conclusions

In the present study, the chloroplast genome of snap bean was sequenced and assembled, which exhibited a conserved quadripartite structure and typical AT-rich plastomic features shared with other legumes. Phylogenetic analysis revealed that snap bean is genetically closest to dry common bean, and the genus *Phaseolus* is phylogenetically sister to *Vigna*. Four highly divergent plastid hotspots (accD, trnY-psbD, ndhH and ycf1-rps15) were identified as promising candidate molecular markers for future phylogenetic and population genetic studies, while the genes *accD* and *ycf2* were found to bear signatures of positive selection. The plastomic resources advance our understanding of chloroplast evolution in common bean and will aid future work in legume genetics, phylogenomics, and crop improvement.

## Figures and Tables

**Table 1:** Base composition of the *Phaseolus vulgaris* L. (snap bean) chloroplast genome.

Region	A (%)	G (%)	C (%)	T (%)	G + C (%)
LSC	33%	16%	17%	34%	33%
SSC	37%	15%	13%	35%	28%
IR	29%	22%	20%	29%	42%
Total	32%	17%	18%	33%	35%

**Table 2:** The list of genes in the chloroplast genome of snap bean.

Classification	Genes
Light dependent photosynthesis	
Photosystem I	*psaJ*, *psaI*, *psaB*, *psaC*
Photosystem II	*psbH*, *psbN*, *psbT*, *psbB*, *psbE*, *psbF*, *psbL*, *psbJ*, *psbK*, *psbI*, *psbM*, *psbD*, *psbC*, *psbZ*, *psbA*
NADH dehydrogenase	*ndhJ*, *ndhK*, *ndhC*, *ndhB* ^a^(2), *ndhH*, *ndhA* ^a^, *ndhI*, *ndhG*, *ndhE*, *ndhD*, *ndhF*
Cytochrome b/f complex	*petD* ^a^, *petB* ^a^, *petG*, *petL*, *petA*, *petN*
ATP synthase	*atpA*, *atpF* ^a^, *atpH*, *atpI*, *atpE*, *atpB*
Large subunit of Rubisco	*rbcL*
Function uncertain	*ycf1*, *ycf2*
Self-replication	
Proteins of large ribosomal subunit	*rpl36*, *rpl20*, *rpl14*, *rpl16* ^a^, *rpl2* ^a^(2), *rpl23*(2), *rpl32*
Proteins of small ribosomal subunit	*rps8*, *rps11*, *rps12* ^b^(2), *rps18*, *rps2*, *rps14*, *rps3*, *rps19*(2), *rps7*(2), *rps15*
Subunits of RNA polymerase	*rpoA*, *rpoC2*, *rpoC1* ^a^, *rpoB*
Ribosomal RNAs	*rrn16S*(2), *rrn23S*(2), *rrn4.5S*(2), *rrn5S*(2)
Transfer RNAs	*trnP-UGG*, *trnW-CCA*, *trnQ-UUG*, *trnS-GCU*, *trnG-UCC* ^a^, *trnR-UCU*, *trnC-GCA*, *trnD-GUC*, *trnY-GUA*, *trnE-UUC*, *trnT-GGU*, *trnS-UGA*, *trnG-GCC*, *trnfM-CAU*, *trnS-GGA*, *trnT-UGU*, *trnL-UAA* ^a^, *trnF-GAA*, *trnV-UAC* ^a^, *trnM-CAU*, *trnK-UUU* ^a^, *trnH-GUG*, *trnI-CAU*(2), *trnL-CAA*(2), *trnV-GAC*(2), *trnI-GAU* ^a^(2), *trnA-UGC* ^a^(2), *trnR-ACG*(2), *trnN-GUU*(2), *trnL-UAG*
Other genes	
Maturase	*matK*
Protease	*clpP* ^b^
Envelope membrane protein	*cemA*
Acetyl-CoA carboxylase	*accD*
c-type cytochrome synthesis gene	*ccsA*

^a^ Gene with one intron; ^b^ gene with two introns; gene(2) number of copies of multi-copy genes.

## Data Availability

The assembled chloroplast genome is publicly available in GenBase (https://ngdc.cncb.ac.cn/genbase/, accessed on 19 September 2026) under accession number C_AA101375.1.

## Supplementary Materials

The following supporting information can be downloaded at https://www.mdpi.com/article/10.3390/ijms27188393/s1.
