# Supplementary material for: Chloroplast Genome Sequence of Snap Bean (Phaseolus vulgaris L.) and Comparative Analyses with Other Legume Chloroplast Genomes
Source: Int J Mol Sci. 2026 Sep 20;27(18):8393. doi: 10.3390/ijms27188393 (PMC13607189; doi:10.3390/ijms27188393)
Supplement: Supplementary file 1 [file ijms-27-08393-s001.zip › Supplementary Figures.pdf]

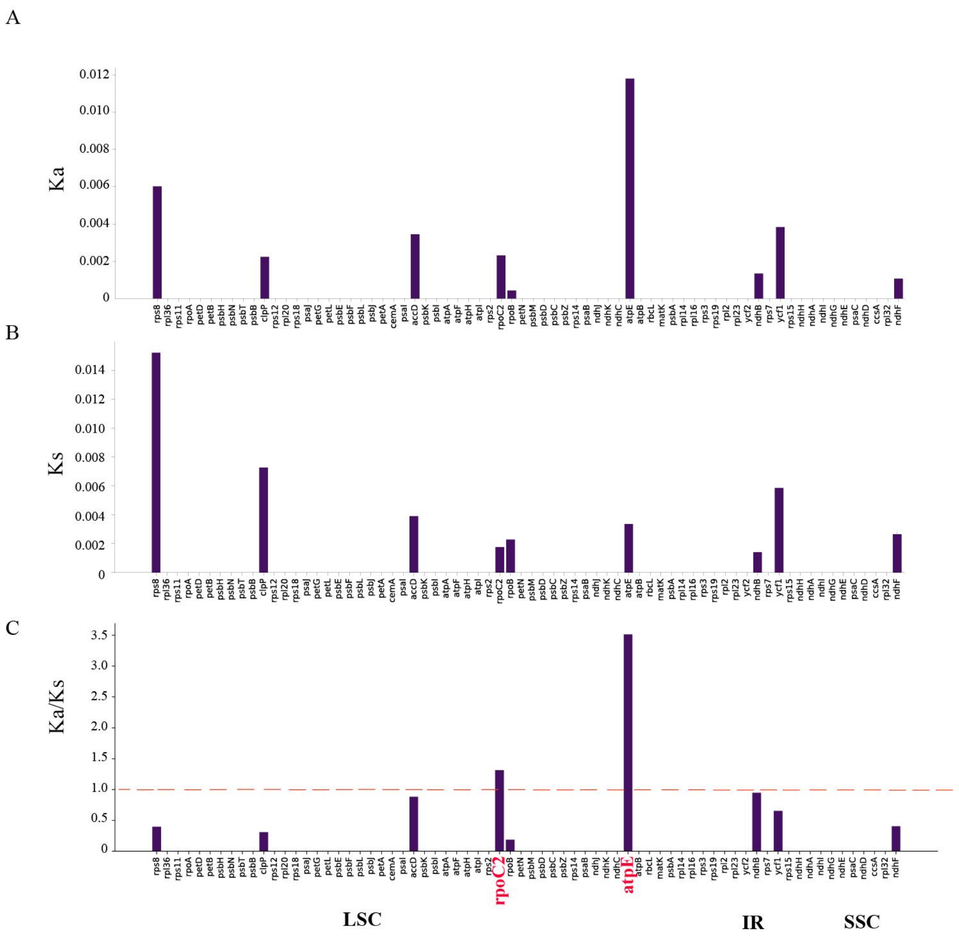

Supplementary Figure S1. The Ka, Ks and Ka/Ks value between snap bean and dry bean.
